# Can a Robot Catch You Lying? A Machine Learning System to Detect Lies During Interactions

**DOI:** 10.3389/frobt.2019.00064

**Published:** 2019-07-31

**Authors:** Jonas Gonzalez-Billandon, Alexander M. Aroyo, Alessia Tonelli, Dario Pasquali, Alessandra Sciutti, Monica Gori, Giulio Sandini, Francesco Rea

**Affiliations:** ^1^RBCS, Istituto Italiano di Tecnologia, Genova, Italy; ^2^DIBRIS, University of Genova, Genova, Italy; ^3^CONTACT, Istituto Italiano di Tecnologia, Genova, Italy; ^4^UVIP, Istituto Italiano di Tecnologia, Genova, Italy; ^5^ICT, Istituto Italiano di Tecnologia, Genova, Italy

**Keywords:** deception, ocular behavior, humanoid robot, lie detection, random forests

## Abstract

Deception is a complex social skill present in human interactions. Many social professions such as teachers, therapists and law enforcement officers leverage on deception detection techniques to support their work activities. Robots with the ability to autonomously detect deception could provide an important aid to human-human and human-robot interactions. The objective of this work is to demonstrate the possibility to develop a lie detection system that could be implemented on robots. To this goal, we focus on human and human robot interaction to understand if there is a difference in the behavior of the participants when lying to a robot or to a human. Participants were shown short movies of robberies and then interrogated by a human and by a humanoid robot “detectives.” According to the instructions, subjects provided veridical responses to half of the question and false replies to the other half. Behavioral variables such as eye movements, time to respond and eloquence were measured during the task, while personality traits were assessed before experiment initiation. Participant's behavior showed strong similarities during the interaction with the human and the humanoid. Moreover, the behavioral features were used to train and test a lie detection algorithm. The results show that the selected behavioral variables are valid markers of deception both in human-human and in human-robot interactions and could be exploited to effectively enable robots to detect lies.

## 1. Introduction

Deception is a complex skill that takes part in human social interactions and can be achieved by different means. According to Mahon (2016) lying can be defined as the act of hiding the truth using a false statement with the intention to make someone else believe it.

In modern society, lie detection has a relevant impact on social activities, particularly on those which require tutoring (e.g., in education or healthcare), being a necessary skill for a broad range of professions such as teachers, doctors, or law enforcement officers. Those individuals are usually trained to detect lies but it has been proven that their ability to differentiate between liars and truth teller is often imprecise. For example, Vrij (2004) reported that experienced professionals such as police officers have an average accuracy of 65% when asked to detect lies and does not depend on precise behavioral cues known to be associated with lying, but rather on subjective experience.

Traditional methods for lie detection involve the use of multiple devices such as polygraph, sweat and respiratory rate measurement, heartbeat sensor and blood pressure monitor. The most common method used, the polygraph reach high accuracy in detecting lies ranging from 81 to 91% (Gaggioli, 2018). However, they are invasive and require an experienced human interviewer to conduct the interrogation and interpret the results. Moreover, those techniques are not always reliable, as it has been demonstrated that trained people can have high success to trick the system (such as the polygraph Honts et al., 1994). For these reasons, they cannot be implemented in robotic systems. On the other hand, there are new scientific findings showing that more objective indicators can be related to lie behavior. Indeed, recently, some behavioral cues such as eye movements and speech have gathered considerable attention as relevant lying indicators which could be easily portable on autonomous systems and could reduce the invasiveness of the process. In two different studies, (Honts et al., 1994; DePaulo et al., 2003) showed that lying could require more cognitive load compared to truth telling. For example, liars need to build a plausible and coherent story, which would increase their cognitive load (Kassin, 2005) and eye blinking and pupil dilation are usually associated to a higher cognitive load (Stern et al., 1984). In particular, Beatty and Lucero-Wagoner (2000) identified three useful task-evoked pupillary responses (TEPRs): mean pupil dilation, peak dilation, and latency to the peak. Another example of the importance of the pupillary response has been provided by Dionisio et al. (2001). In the study, they asked students to reply to questions, sometimes by telling the truth or by lying. The task-evoked a significantly greater pupil dilation when participants were confabulating responses compared to when they were saying the truth about an episodic memory. These results suggest that the increase of the pupil size could be associated with the production a deceiving behavior. Recent evidence in the literature from Leal and Vrij (2008), Webb et al. (2009), Walczyk et al. (2012), and Vrij et al. (2015) propose a direct link between lie creation and oculomotor patterns such as blinking, fixations, saccades, and pupillary dilation.

Together with the study of oculomotor patterns, speech features has been demonstrated to be relevant to distinguish deceptive behaviors. Hung and Chittaranjan (2010) investigated acoustic prosodic features such as pitch, energy, F0 measurement and speaking rate as possible markers to detect liars. Mihalcea and Strapparava (2009) explore the detection of liars leveraging on specific language use. They annotated a corpus of text of truth and lie statements and characterize class of words that could be discriminant to differentiate truth to lie statements. Hirschberg et al. (2005) combine both of the aforementioned features to detect deceptive behaviors with an accuracy that was just above their baseline (predicting in majority lies). While this studies are promising (Hirschberg et al., 2005) shows that leveraging on these features can be powerful markers of deception when specifically tailored to the speaker. Levitan et al. (2016) also found that phonotactic features could not generalize well to new speakers suggesting that these features are also speaker dependent. Further, time to respond to questions during an interrogation has been identified as another speech related variable and a possible cue of deceptive behavior (Seymour et al., 2000). Walczyk et al. (2003) tested whether elaborating deceptive answers could be correlated to the time to respond. They discovered that the decision to lie increments the time to respond, especially in open-ended questions (i.e., questions that elicit more than two possible answers).

The proposed lie detection system is based on the cognitive framework on deception that lying comes with a higher cognitive cost. This assumption is supported by the finding that lying typically takes longer than truth telling (Walczyk et al., 2003). Among all the considered speech features associated with deception, we choose to consider the time to respond in our lie detection model as it has been proven to be a marker of cognitive load.

Considering all the results from previous studies on the physiological variables associated with deception, a possible solution to the lack of precision in the detection of lies can come from the use of new technologies in terms of artificial intelligence and social robotics.

Nowadays, robots are starting to be used in the context of professional activities that require social skills, such as security, education, or healthcare (Basoeki et al., 2013; Robinson et al., 2014). Therefore there is a need for developing social robots capable of understanding when humans are deceitful with them to a similar extent of how humans do. Robots can leverage on sensors and computational capabilities to monitor specific behavioral cues that can be used to identify when a person is lying. Eyes movements and speech features can be monitored by minimally-invasive sensors, an eye tracker device and a microphone can be used to process them and can be integrated into a robotic platform without requiring a complex setup.

In this work, we hypothesize that the use these behavioral cues associated with deception already investigated in human-human interaction (HHI) could be used to develop a lie detection system using a humanoid robot, with a noninvasive measurement approach. To verify our hypothesis, first, we measured the participants' behavioral responses to evaluate whether there are differences between lying to a human interviewer or to a humanoid robot. An interrogatory scenario was designed, in which participants were told that they had been witnesses of a crime and they should lie to protect the perpetrators or tell the truth to bring them to justice. The iCub humanoid robot (Metta et al., 2008), and a human confederate played the role of interviewers. As variables of evaluation of the behavior, eye movements, time to respond and eloquence were taken into account, which, as discussed above, vary according to the type of deceptive behavior.

As hypothesized, we observed the same behavioral pattern between liars and truth tellers when the interviewer was a robot or a human. After this verification, we tested whether a learning algorithm can be trained to detect lies starting from the behavioral features highlighted in the first study. Each question gathered during the experiment was used to create a data set on which a random forests algorithm was trained to label the reply as truth or lie. Then the model was tested to assess the ability to generalize to novel questions and to novel interviewees.

## 2. Materials and Methods

### 2.1. Participants

28 participants with an average age of 24.5 years (*SD* = 5.31) took part of the experiment. All of them were Italian speakers, 9 males and 17 females with a broad educational background; they all received a monetary reward of 15 euros to participate in the experiment. All of them signed an informed consent form approved by the ethical committee of Liguria region, in which, it was stated that camera and microphone could record their performance, and agreeing on the use of their data for scientific purposes.

### 2.2. Procedure

The experiment is inspired from the work of Walczyk et al. (2012) where participants are asked to tell the truth or to lie based on two crime videos (see Supplementary Material) they had been “witnesses.” Participants were asked to lie to protect one of their relative that committed a robbery by misleading the interrogators. Participants were evenly distributed among a 2 × 2 × 2 conditions to avoid any ordering effects (agent: human or robot investigator; witness: truth-teller or liar; and two different videos). The agent order was kept constant within the same condition. The experiment was divided into three phases: (i) general questions, (ii) first session of question, (iii) second session of question. This protocol is a modified version of a previous pilot study of Aroyo et al. (2018a). Participants were asked to fill a psychological questionnaire prior to the experiment and a post-interview questionnaire after the experiment.

#### 2.2.1. Phase One

**Figure 1:**
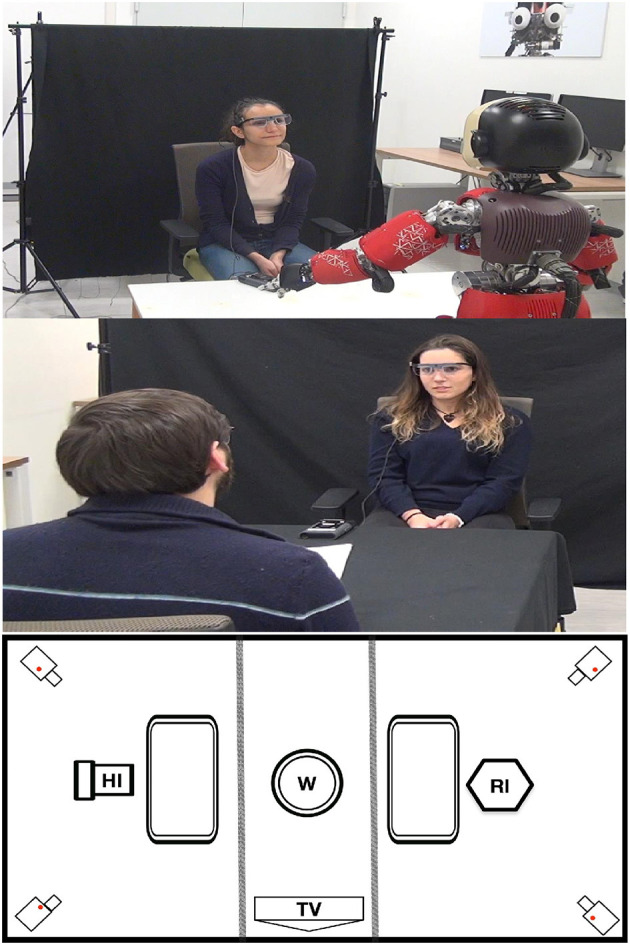
Experimental setup: **(Top)** robot investigator (RI) debriefing a witness (W); **(Middle)** human investigator (HI) debriefing a witness (W); **(Bottom)** interrogation room display (Informed consent of participants has been obtained for the use of their photo).

After giving their consent to take part to the study, participants were welcomed by an experimenter, who explained the general purpose of the experiment:“You have been witnesses of two crimes, and you have to help the investigators to find out the responsible.” Once in the room, the experimenter asked participants to wear the Tobii Eyetracker glasses and Polar H10 heart-rate chest sensor. The experimenter asked the participant to sit in the middle of the room (Figure 1) and calibrated the Tobii glasses. Then participants were instructed to answer truthfully and quickly to 20 general questions (e.g., “Can an oven be hot?”, “What is the first name of Berlusconi?”). Respectively the first 10 questions were asked by the robot investigator and the remaining 10 by the human investigator (Figure 1-top, middle). The order of the block of human and the robot questions was alternated within the participants. In the room a black curtain separated the participant and the investigator from the inactive investigator. The experimenter always left the interrogation room before the investigator started the questions.

#### 2.2.2. Phase Two

After completing the first phase of general questions, the experimenter entered the interrogation room and gave participants an instructions sheet. It was written that they will see a video surveillance of a crime and pick “randomly” a role from a box (the randomization was just to give the impression of choice since the role was defined a priori). Inspired by Walczik et. al, the role could either be: truth tellers - a witness who wants the criminals brought to justice, thus, to reply to all the questions truthfully; or protectors a witness who realized that the criminal is a familiar of theirs, therefore they should lie to all the questions in order to protect the familiar. In both cases, the experimenter specified that their financial award will vary according to their performance in keeping the assigned role (telling the true or lie). Participants watched a video of a crime, after that the experimenter put and calibrated the Tobii glasses again, and left the interrogation room to the investigators. Either the robot or human, asked 10 questions each, in the two different locations of the room (Figure 1-bottom). The investigators made two types of questions: short type - yes/no questions, and open-ended questions. These questions differ in syntactic constraints that put on permissible responses (Walczyk et al., 2013). An example of a short question is “Did it happen in a crowded place?”; while an open-ended one is “Describe what the suspect was wearing”.

#### 2.2.3. Phase Three

After the end of the second phase, the experimenter entered in the room again and made the witness pick “randomly” a role (the condition was forced to be the opposite of the previous one). It was repeated that they will be rewarded according to their performance in keeping the role that they were assigned. Then similarly as in phase two participants watched the second video and once finished the experimenter calibrated the Tobii glasses and leave the room. As in phase two the interrogators asked 10 questions each (5 close question and 5 open-ended). When both interrogators finished, the experimenter entered in the room to remove the glasses, and to ask participants to compile a final questionnaire before finishing the experiment. At the end of the experiment, the experimenter removed the heart-rate chest sensor and accompanied the participant to leave the room. Finally, participants were debriefed, received their compensation and could ask clarifications to the experimenter about the experiment.

### 2.3. Design and Tasks

The experiment was design as a within subject, with the following two conditions: truth-teller and liar. The dependent variables were eloquence (time of speaking), time to respond, number of saccades, number of fixations, number of blinks, average pupil diameter, maximum pupil diameter, minimum pupil diameter. All participants filled a psychological profile questionnaire to estimate their personal traits:: Big five (extraversion, agreeableness, conscientiousness, neuroticism, openness) (Flebus, 2006), Negative Attitude toward Robots Scale(NARS) (Syrdal et al., 2009), histrionic (Ferguson and Negy, 2014), narcism machiavellism (Jones and Paulhus, 2014).

The experiment setup was prepared as an interrogation room (Figure 1, bottom). The room was divided into three zones separated by black curtains, with the witness (W) seated in the center on a rotating chair. This setup allowed to quickly switch from robot (RI) to human interviewer (HI) and to ensure complete isolation during the interrogation. Two cameras, 4K and HD, were used to record the participants when they were interviewed and where placed at the four corners (Figure 1). Moreover, it was asked to each participant to wear a heartbeat sensor and an ambient microphone. The recording of the heartbeat and the cameras were not analyzed, but only used to create a more realistic interrogatory scenario.

### 2.4. Measurements

#### 2.4.1. Eyes Movements

Participant's eyes movements were recorded using the Tobii Eyetracker glasses (Tobii Pro Glasses 2). These glasses were used to record pupil dilation and eyes movements, with a frequency of 100Hz. All eyes features were computed within the temporal window defined by the start of the question until the stop of the answer as in Webb et al. (2009).

To ensure same setup for all the participants during different times of the day, the window blinds were closed, and the room was lit with artificial light. Moreover, the luminance of the room between the locations of investigators (Figure 1) was measured. The percentage change of LUX values between the two locations is about 0.25% indicating that the luminosity was the same for both locations and did not influence the pupillary measurements. This guaranteed the minimum variability in the pupil measurements from external factors.

#### 2.4.2. Time to Respond, Eloquence

Speech features such as the time participants took to respond to the questions and the time they spent speaking were manually annotated from the audio extracted from the Tobii Eyetracker using the open-source software Audacity. Each question was annotated using the following four tags: start question (STAQ), stop question (STOQ), start answer (STAA), stop answer (STOA). We calculated the start tags (STAQ, STAA) by considering the first word uttered and for the stop tags (STOQ, STOA) the last one. All tags were annotated with a precision of milliseconds. With these tags we computed the time to respond as the temporal difference between the STOQ and STAA. Eloquence was defined from the temporal difference of the tag STOA and STAA.

#### 2.4.3. Automatic Processing of Speech Features

We investigated the automatic annotation of the different tags (STAQ, STOQ, STAA, STOA) leveraging on a state of the art voice activity detector (VAD) WebRTC VAD (Google, 2015). We used it to detect the different speech tags on segmented parts of the audio of the participant interview. The segmented parts were calculated by taking the start of the question until the next start of the question. As the final goal of this study is to propose an approach to allow to detect lies with a humanoid robot we assumed that the tags start of the question (STAQ) could be known from the speech synthesizer used on the robot. This allowed us to segment all the different interview sessions with the robot in several cropped audio file on which the VAD was run. We then compared the outputs of the VAD on different random participants with the manual annotation. We compute the difference by calculating the mean square error. We can see that the algorithm get reasonable results but struggle with some annotation like with participant s8 in the general condition (Table 1). This could be explain by the speech dynamic of some of the participants, indeed some of the participants produces sound before actually answering to the questions. This produced false positives in comparison to the manual annotation as we consider the first and last word uttered to compute the tags. In regards of the results of the automatic annotations we choose to consider for the rest of the paper the manually tagged speech features.

**Table 1:** Mean square error between the ground truth (manual annotation) and automatic annotation on the debriefing with the robot in different conditions.

**Participant**	**Conditions**	**Mean square error**
s0	video1	51.83
s4	video2	122.86
s19	video2	47.52
s1	video2	95.81
s8	baseline	170.50
s12	video2	10.42
s16	video1	3.99
s21	video2	77.0

## 3. Results

Two participants had to be removed, one because the participant knew the human investigator and it could have influenced her behavior during the experiment. The second was removed because of experimenter's error (the participant was assigned the same role, truth teller, for both videos).

### 3.1. Behavioral Cues Human vs. Robot Investigator

The first hypothesis addressed in this paper is to assess the possibility to use a humanoid robot as an interviewer during an interrogatory scenario. A 3-way repeated measure ANOVA^1^ was performed with the three factors: veracity (truth teller, liar); agent type (human, robot); question type (short, open-ended). The behavioral responses analyzed were: number of fixations, number of blinks, number of saccades, average pupil dilation (left and right), maximum and minimum pupil dilation (left and right), eloquence and the time to respond.

Considering the factor agent type a statistical significance was found in the number of fixations: participants tend to fixate more the robot interviewer with respect to the human interviewer independently of the question type [*F*_(1, 24)_ = 4.92, *p* < 0.05]. The average pupil dilation for both the left and right eye [*F*_(1, 24)_ = 41.36, *p* < 0.001; *F*_(1, 24)_ = 42.93, *p* < 0.001] and the minimum pupil dilation [*F*_(1, 24)_ = 41.69, *p* < 0.001; *F*_(1, 24)_ = 44.73, *p* < 0.001] were found to be significantly different. The average and minimum pupil response was higher when people interact with the human interviewer in comparison of a robotic interviewer.

Interestingly, no statically significance was found for eloquence [*F*_(1, 24)_ = 0.84, *p* = 0.36], and time to respond [*F*_(1, 24)_ = 1.26, *p* = 0.27]. The same pattern was observed between the robot and human investigator. Participants spoke more when telling the truth when responding to open-ended questions and always took more time to answer when lying.

This tendency was confirmed by the 3-way ANOVA analysis on the experimental data between truth tellers and liars. The ANOVA analysis showed a statistically significant for the veracity factor and speech features : eloquence [*F*_(1, 24)_ = 7.25, *p* = 0.01],time to respond [*F*_(1, 24)_ = 25.59, *p* < 0.001]. Confirming that participants when telling a lie take more time to respond for both short and open-ended questions but tend to speak more only when they have to answer truthfully to open-ended questions (Figure 3).

For eyes features a statically significance was found for the number of saccades [*F*_(1, 24)_ = 4.26, *p* = 0.02], the average pupil dilation [*F*_(1, 24)_ = 7.11, *p* = 0.01] and the minimum pupil dilation [*F*_(1, 24)_ = 5.84, *p* = 0.02]. As demonstrated in the literature deceptive people exhibit a higher pupil response than truth tellers.

The participant's psychological profile was analyzed by running a Pearson correlation analysis on all behavioral cues to check whether specific psychological factors relate to specific behavioral responses. None of the psychological factors were correlated with any of the behavioral cues responses.

**Figure 2:**
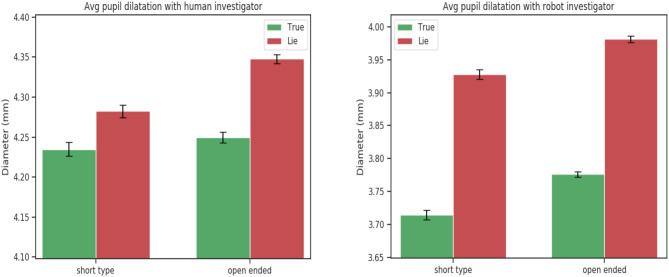
Mean left pupil dilation for short and open-ended questions. Statistically significant for factor agent type [*F*_(1, 24)_ = 41.36, *p* < 0.001] and veracity [*F*_(1, 24)_ = 5.84, *p* = 0.02].

**Figure 3:**
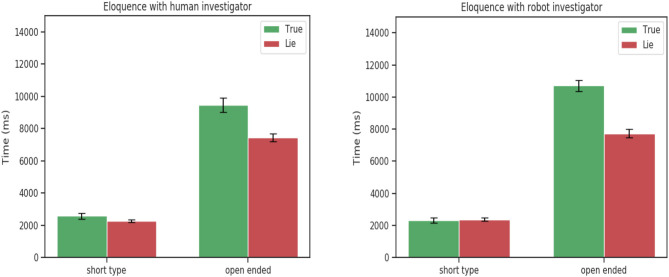
Eloquence for short and open-ended questions. Statistically significant for factor veracity [*F*_(1, 24)_ = 7.25, *p* = 0.01].

**Figure 4:**
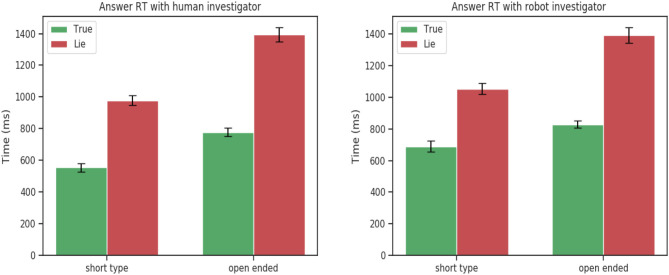
Time to respond for short and open-ended questions. Statistically significant for factor veracity [*F*_(1, 24)_ = 25.59, *p* < 0.001].

These results comforted our hypothesis that a humanoid robot can be used as an interviewer to detect lies to the same extent of a human interviewer. While there exist significant differences between the interaction with a human and robot, mainly in the eyes features (fixations, pupil dilation). These differences are unrelated with the deceptive or truthful behaviors of participants and same patterns are observed when the interaction is with a robot or a human (Figures 2–4). The significant behavioral responses (fixations, average and minimum pupil dilation) were not significant when considering the double factor agent and veracity [*F*_(1, 24)_ = 1.18, *p* = 0.28, *F*_(1, 24)_ = 0.002 *p* = 0.96, *F*_(1, 24)_ = 0.72, *p* = 0.40 ].

### 3.2. Learning to Detect Lies

The second objective addressed in this paper is to assess whether a learning algorithm could be trained to detect lies from these behavioral features. According to previous statistical analysis, leveraging on eyes movements and speech features would promise good results. To build the lie detector system a data set was created from the behavioral response of the participants to the experiment. The data set is composed of the collection of measurements of behavioral response to questions classified as truths or lies.

Consequently, the problem can be defined as a binary classification with the input attribute vector *X* (behavioral features) and the target vector *Y* ∈ [0: *True*; 1: *Lie*].

To build the lie detector an ensemble learning method based on decision trees was chosen : random forests (Breiman, 2001). Random forests have desirable characteristics, they tend to not overfit when increasing the number of tress, and they can deal with mix of categorical and non-categorical features (Breiman, 2001). Moreover, normalization is not required for random forests because each node in the tree is simply splitting a sorted list and not comparing one feature's value to another feature's value. This allow to used the trained model without requiring heavy pre-processing. For these reasons, random forests are a good candidate to address this classification problem.

#### 3.2.1. Features Selections

The literature (Walczyk et al., 2003, 2012; Webb et al., 2009) and experimental results on lie detection demonstrate that eyes features and speech features have been shown to be significant to discriminate between lies and true statements. Eyes features including: pupil dilation, number of saccades, fixations and blinks are known markers for detecting deception. The statically analysis run on the experimental data confirms the significance of the number of saccades, the average and minimum pupil dilation, the eloquence and the time to respond between liars and truth tellers. The analysis further shows that the question type was statically significant for all the behavioral features. In order to take into account this effect a categorical variables question type was introduced. Moreover, the psychological traits were used along with the behavioral cues.

Leading to two different sets of attributes tested in this classification problem: (i) *X*_1_ (Eloquence, Time to respond, Average pupil dilation left, Minimum pupil dilation left, Number of saccades and Question type) (ii) *X*_2_ (*X*_1_ and psychological profile).

#### 3.2.2. Classification Result

It was first investigated if an universal lie detector could be trained from the experimental data gathered. Given these two attribute vectors *X*_1_ and *X*_2_, the data set was split between training and testing sets by taking 80% of the participants for training and the remaining 20% for testing, it is further referred to this specific classification as a between generalization.

A grid search method with cross-validation was run to find the optimal values of the hyper parameters for the random forests algorithm. The desirable model should be trained to minimize type II error (false negative) to miss the less possible lies while providing a good precision. It is preferable to identify that a person is lying, refining afterward whether it was a false positive, rather than to miss a lie, which could have worse consequences. Consequently, the model was optimized to maximized the recall score to minimize missing lies.

Due to the relative low size of the data gathered during the experiment (20 questions for label and interviewers) it was considered rational to merge the two sets of interviewers' data : the robot and the human interviewer data. Justification of such approach is in the fact that the ANOVA analysis showed no statistical differences for the double interaction agent type and veracity for all behavioral features considered in this classification problem. It was then chosen to consider all the data independently from the interviewer leading to a data set of 1,014 questions (510 lies, 504 true statements). Results of the best model found with cross-validation with the two set of attributes are reported in Table 1. Several metrics beyond accuracy were analyzed such as the number of true positives (TP), true negatives (TN), false positives (FP), and false negatives (FN) to have a better understanding of the performances of the models. Moreover, the AUCROC score (Fan et al., 2006) is reported which gives a good metric to test the separability power of the model independently of the classification threshold.

The best model trained with the behavioral features achieved an accuracy of 69% and an AUCROC score of 0.74. Looking at the misclassification errors, it can be seen that the model is sensitive detecting 82% lies correctly but with a precision of 65%. Surprisingly adding the psychological traits decreased the performance of the model with a drop of accuracy and precision but a marginal gain in sensitivity.

To further test the generality of the proposed model, it was evaluated on the data from an additional sample of participants who were presented with a different set of questions [derived from the pilot study (Aroyo et al., 2018a) composed of 547 questions (276 lies, 271 true statement)]. From the previous studies, improvements to the protocol of the experiment have been made due to the following main issues encountered: (i) lack of talk from participants, (ii) goodness of the lies. To address these changes, the protocol has been changed to motivate participants to lie through monetary reward. The second change was motivated by the results of the previous study where open-ended question did not elicit long answers in participants. The open-ended questions were changed by using specific words to force participants to talk such as describe me, “tell me.” Validating our model with similar data collected in different experiment could give us a meaningful insight on how the system could generalize. The psychological profile was not used as the questionnaires used differed from the pilot study, results are reported in Table 2.

**Table 2:** Lies classification results, between generalization (robot + human merged).

**Set of attributes**	**TP**	**TN**	**FP**	**FN**	**Recall**	**Precision**	**Accuracy**	**AUCROC score**
*X*_1_	82	56	44	18	0.82	0.65	0.69	0.74
*X*_2_	89	39	61	11	0.89	0.59	0.64	0.76

The model trained with the set of attributes *X*_1_ achieved an accuracy of 63% with an AUCROC score of 0.69. While the performance dropped, the model was still able to detect 68% of the lies but produced many false positives (114).

Since the main objective of the current study was to demonstrate that a robot could be use to detect lies, we investigated how the model performance varies when data were extracted according to the agent type. Classification performances was compared in the conditions where the model was trained on data associated with the two different agents: (robot vs. human). The previous data set was subdivided according to the agent type leading to (i) Set of questions associated with robot investigator (503 instances, 255 lies and 248 true) (ii) Set of questions associated with the human investigator (511 instances, 255 lies and 256 true). Table 3 summarizes the results obtained for the best models found with cross-validation for both sets of attributes *X*_1_ and *X*_2_.

**Table 3:** Lies classification results, between generalization (robot + human merged) tested on pilot study data.

**Set of attributes**	**TP**	**TN**	**FP**	**FN**	**Recall**	**Precision**	**Accuracy**	**AUCROC score**
*X*_1_	189	158	114	87	0.68	0.62	0.63	0.69

The best model trained with behavioral cues on the robot data set achieved an AUCROC score of 0.76 with an accuracy of 65%. The model was able to detect 88% of the lies but with a precision of 60%. In comparison, the best model trained with human data performed better with an accuracy of 69% but with a lower AUCROC score of 0.74. The model was less sensitive with a recall value of 78% but produce less false positives with a precision value of 66%. Adding the psychological traits improved marginally the classification performance on data associated with the human interviewer and decreased for data associated with the robot interviewer. It is worth noticing that in both cases psychological traits allow to augment the sensitivity of the models. As the previous analysis, both models were evaluated with the pilot study data by taking into account the agent type. Only the set of attributes *X*_1_ was considered, results are reported in Table 4.

**Table 4:** Lies classification results, between generalization, considering the agent type.

**Agent**	**Set of attributes**	**TP**	**TN**	**FP**	**FN**	**Recall**	**Precision**	**Accuracy**	**AUCROC score**
Robot	*X*_1_	44	21	29	6	0.88	0.60	0.65	0.76
Human	*X*_1_	39	30	20	11	0.78	0.66	0.69	0.74
Robot	*X*_2_	47	16	34	3	0.94	0.58	0.63	0.74
Human	*X*_2_	44	27	23	6	0.88	0.66	0.71	0.76

Both models performances remain quite stable with a marginal drop of accuracy in comparison of the previous evaluation on the experimental data. Looking inside the misclassifications errors, the precision remain the same for the robot data and increased for the human data. For both models, the drop of performances was mainly attributed to the decrease of sensitivity.

While between generalization is appealing and could be applied in diverse work case scenarios, the classification task is more complex has the model has to generalized and learn generic rules that applied to every type of persons. Another type of generalization that could fit a different use of a robot lie detector is a within generalization. In healthcare, educational scenarios, usually the group of persons monitored remain constant. Therefore, instead of a building an universal lie detector, it could be interesting to consider a lie detector trained for a specific set of persons to infer if their novel affirmations are true or false. To address this new possible use case a new data set was created by taking for each participants and for each conditions (true, lie) seven questions for training and three for testing. The same procedure has before was followed, first the data were merged and later separated between agent type to compared the performance when considering agent type, results as reported in Tables 5–7. As previously the psychological traits improved marginally the classification results it was chosen to ignore them for the within generalization.

**Table 5:** Lies classification results tested on pilot study data, between generalization (271 questions for robot interviewer, 276 for human interviewer.

**Agent**	**Set of attributes**	**TP**	**TN**	**FP**	**FN**	**Recall**	**Precision**	**Accuracy**	**AUCROC score**
Robot	*X*_1_	106	65	70	30	0.78	0.60	0.63	0.69
Human	*X*_1_	84	104	32	56	0.60	0.72	0.68	0.74

**Table 6:** Lies classification results, within generalization (robot + human merged).

**Set of attributes**	**TP**	**TN**	**FP**	**FN**	**Recall**	**Precision**	**Accuracy**	**AUCROC score**
*X*_1_	55	59	19	23	0.71	0.74	0.73	0.77

**Table 7:** Lies classification results, within generalization considering the agent type.

**Agent**	**Set of attributes**	**TP**	**TN**	**FP**	**FN**	**Recall**	**Precision**	**Accuracy**	**AUCROC score**
Robot	*X*_1_	27	27	14	10	0.73	0.69	0.69	0.76
Human	*X*_1_	23	29	8	18	0.56	0.74	0.66	0.68

Considering independently the agents, the best model achieves an accuracy of 73% with an AUCROC score of 0.77. The model was able to detect 71% of the lies with a precision of 74% producing relatively low false positives. Taking into account the agent type, both classification performances dropped, but differently from the between generalization, the data associated with the robot interviewer achieved the best performances.

## 4. Discussion

In this work, we hypothesize that a humanoid robot could be used as a lie detector leveraging on known behavioral cues present human-human interactions associated with deception. We brought evidences that our hypothesis was correct. To verify it, firstly, we showed that there were no difference between the behavioral indicators associated with lie detection when the interaction is between a human and a human and a human and a robot interviewer. Secondly, we trained a machine learning model to detect lies demonstrating that a robotic system can be used for lie detection during human-robot interaction.

The research purpose was addressed by designing an HRI scenario where participants had to lie or tell the truth on questions based on a crime they were witnesses. The behavioral cues recorded during the experiment were analyzed with three way repeated measure ANOVAs to investigate any statically difference between the reactions to a human and a humanoid robot investigator. The results shows that only features associated with eyes (average pupil dilation, fixations and minimum pupil dilation) change significantly as a function of the agent nature. The differences in the eyes or speech features between false and true responses were however similar during human-human and human-robot interaction (no significant interactions between veridicality and agent-type). In particular the time to respond, the eloquence (i.e., the duration of the response), number of saccades and the average and minimum pupil dilation were different between true and false replies. These results demonstrated the viability of using a humanoid robot as interviewer. Indeed, the difference of magnitude for the average and minimum pupil dilation in presence of a robot interrogator compared to a human interviewer has no influence on the ability to discriminate between liars and truth tellers.

Supported by these results, it was tested if a machine learning algorithm could be trained on the experimental data to build a lie detector. The preferred machine-learning algorithm chosen was a random forests. The first generalization explored how to train a model to detect lies generated by new persons, to build an universal lie detector. To address this, a subset of participants was taken to train the model and the others for testing. It was first considered the full data set independently of the agent type with two different set of attributes (eyes, speech features and psychological traits). The best model achieved an accuracy of 69% and an AUCROC score of 0.74 (Table 1). Looking at the misclassifications errors, it can be seen that the model detected 82% lies correctly but with a precision of 65%. Surprisingly, adding the psychological traits decreased the performance of the model with a drop of accuracy and precision but promoted a gain in sensitivity. Such result drew us to the hypothesis that the psychological profile does not seems to influence the classification results. Therefore, we focused on eyes and speech features to test further generality of our approach. In the pilot study (Aroyo et al., 2018a) the same markers were monitored which allowed testing the model without adapting the input vector. Moreover, the changes operated on open-ended questions provided a more robust evaluation about the true generality of our approach. The model achieved an accuracy of 63% with a AUCROC score of 0.69. The model was still able to detect 68% of the lies but produced many false positives (114).

It was further investigated how the classification performance will change by taking in account the interviewer type. To address this comparison two additional data sets were created, each one associated with the type of interviewers (robot, human). The best model trained with behavioral cues on the robot data set achieved an AUCROC score of 0.76 with an accuracy of 65%. The model was able to detect correctly 88% of the lies but with a precision of 60%. In comparison, the best model on the human data set performed better in accuracy but with a lower AUCROC score. Adding the psychological traits improved only marginally the classification for the human data set. In addition, for the robot data set it decreased the performance. Looking at the errors made by both models it can be seen that, for both data sets psychological traits increased the sensitivity of the models but decreased the precision for the robot data explaining the drop in accuracy. Similarly as before, the two models were evaluated on the pilot study data. The model trained on human interviewer data set performed better than the one trained with the robot data set with an increased accuracy and AUCROC score (Table 4). Analyzing the misclassifications errors, the model trained with the human data detect less lies than the model with the robot data but produced less false positives. The same differences were found in the two models when tested on the current experimental data and the pilot study. The model trained with the human data tended to produce a more precise classification of lies with fewer false positives. This can suggest that the markers of deception are more discriminant when interacting with a human interviewer rather than when interacting with a humanoid robotic interviewer. Nonetheless, performance achieved with the robot data were encouraging and suggested us to investigate other markers of deception that could be used along the ones used in this study.

Finally, we considered new possible use case scenarios where a lie detector robot would be used to monitor persons such as in a hospital, elderly care, or educational scenario. Investigating how the model could generalize for a group of persons have many practical applications. For example in elderly care the robot will be interacting during months, years with the same group of people, learning their personal traits of deception become then relevant. With these use cases in mind, we looked into training the system to detect lies for a specific set of persons. Considering independently the agents, the best model achieves an accuracy of 73% with an AUCROC score of 0.77. Moreover, it was able to detect 71% of the lies with a precision of 74% producing relatively low false positives. Taking in account the agent type the best performance were achieved considering the robot data set, with an accuracy of 69% and AUCROC score of 0.76. Similarly, to the previous classification comparison between human and robot data, the model trained on human data achieved a higher precision score in detecting lies but with a lower recall value.

However, the proposed method uses cues that can be sensitive to external factors (e.g., eyes' dilation and light conditions), making the portability on real environments more difficult. Moreover, using an eye tracker is a step forward for the development of a less invasive setup that allows the detection of lies but it still requires to be worn and calibrated.

A recent research done by Wangwiwattana et al. (2018) on eye dilation estimation, using RGB cameras, achieved a level of precision as high as the one using standard eye tracker devices. Therefore, it could be used instead of the Tobii device. Furthermore they proposed a technique based on convolutional networks showing robustness to light changes, allowing the record of the pupils in a less controlled environment. Nonetheless, the proposed lie detection system could perfectly be ported in indoor scenarios where lighting can be controlled. Another limitation in our approach is the use of manual annotation rather than leveraging on a automatic methodology. We investigate the accuracy of one of state of the art VAD (Google, 2015) for processing the speech tags and compare it with our manual annotations. Even if we manage to have reasonable results (Table 1) we used the manual annotation to train our machine learning system. Future work could be to train using our data a VAD following the work of Ko et al. (2018) which increased the accuracy compare to VAD (Google, 2015) as well as the speed leveraging on a deep neural network. These two modifications would allow us to extract the same features considered in our paper and used another datasets as the Idiap Wolf Corpus (Hung and Chittaranjan, 2010) as a more generic test for our machine learning system. Another improvements that we think need to be addressed in future work would be to combine acoustic-prosodic, lexical features which has been demonstrated to be valid markers for lie detection (Levitan et al., 2016) with the features considered in our current lie detection system. More recently (Nasri et al., 2016) proposed a lie detection model with an accuracy of 88% leveraging on Mel Frequency Cepstral Coefficient and pitch. These studies make us confident that our current lie detection model could gain in performance by including in this work speech features along the one considered in this work such as the time to respond.

Developing robots capable of detecting lies can have a lot of applications in real life, it endows a robot the capability of understanding whether a person is trustworthy, and it could adapt to that behavior. Apart applying these capabilities in domains such as teaching (understanding whether a child has done their homework), healthcare/homecare (whether patients are taking their meditations) or in law enforcement (spotting possible criminals) is vary valuable in our society. Moreover, they also can be exploited in more negative connotations such as social engineering robots (Aroyo et al., 2018b); or when studying the transfer of authority from a real person to its analog robot (Aroyo et al., 2018c), so to emphasize the strength of the lying cues as the robot could be used to represent a figure with authority such as a teacher, doctor or law officer.

### 4.1. Conclusion

Considering all the classification results, it has been demonstrated that the eloquence, the time to respond, average and minimum pupil dilation, the number of saccades and question type can be used to train a lie detector system. Looking at the misclassifications errors of the different considered models between the robot and human data, interacting with a human interviewer produced better precision in detecting lies. Furthermore, the psychological profile of participants didn't help to improves performances. This can be explained by the population monitored during the experiment, where no extreme psychological traits was found which can grandly influence a behavioral responses. To improve the performance achieved in this study several paths could be considered. The first promotes the acquisition of a bigger data set considered that in this study the data set is rather small (1,054 instances). Furthermore, we speculates that the limitation of the experimental environment could also influence the results. In fact, we believe that in a real interrogatory scenario markers of deception could be even more evident. Improving the data acquisition by designing a better ecological experiment closer to the scenarios in which the robot will operate constitute an important step in the goal of building a lie detector system for a robotic platform.

Additionally, further behavioral or physiological features could be monitored to improve the performance of the proposed system. Iacob and Tapus (2018) recently investigated lie detection in human robot interaction leveraging on non-invasive measurements and found a statically significant difference in the estimated heart beat variability between liars and truth teller. Spatiotemporal features such as the variation of the pupil size trough time or the evolution of the pitch of the voice (DePaulo et al., 2003) may also constitute good candidates to detect lies. Combining these features with the one investigated in this study could lead to advancement in the quest of designing an universal robot lie detector. On the other hand, considering the ability of the system at generalizing to novel responses provided by a participant on which it has been previously trained (within-generalization), the performances achieved demonstrated the possibility to develop a reliable lie detector system.

## Data Availability

The data of the experiment can be asked to the corresponding author (jonas.gonzalez@iit.it).

## Ethics Statement

This study was carried out in accordance with the recommendations of ethical committee of Liguria region with written informed consent from all subjects. All subjects gave written informed consent in accordance with the Declaration of Helsinki. The research protocol was approved by the Regional Ethical Committee (Comitato Etico Regione Liguria), all participants provided their written informed consent and received a compensation of 15 euro.

## Author Contributions

JG-B, AA, and DP run the experiment and analyzed the data. All authors contributed to the design of the experiment and writing of the research article.

### Conflict of Interest Statement

The authors declare that the research was conducted in the absence of any commercial or financial relationships that could be construed as a potential conflict of interest.

## Footnotes

^1^Since a Shapiro test indicated that the eloquence, time to respond, saccades, fixations, blinks, average, minimum pupil diameter left/right, measured during the interviews were distributed non-normally, we tested all this features with a Wilcoxon signed rank tests, which confirmed the reported results. In particular, considering factor Agent we found significance for fixations (*p* = 0.01), average pupil diameter left/right (*p* < 0.001), minimum pupil diameter (*p* < 0.001) all the others features were not statically significant. Considering the factor Veracity we found same significance as with the 3-way ANOVA, eloquence(*p* = 0.003), time to respond (*p* < 0.001), saccades (*p* = 0.020), average pupil diameter left/right (*p* < 0.01). All differences are significant after Bonferroni correction.

## Supplementary Material

Supplementary Material Questions used for the experiments.
