# Supplementary material for: Can a Robot Catch You Lying? A Machine Learning System to Detect Lies During Interactions
Source: Front Robot AI. 2019 Jul 31;6:64. doi: 10.3389/frobt.2019.00064 (PMC7805987; doi:10.3389/frobt.2019.00064)
Supplement: Supplementary Material — Questions used for the experiments. [file Data_Sheet_1.PDF]

Appendix

A. Videos

Below the links of the videos - they have been edited in order to make them shorter.
Video 1: https://youtu.be/w5sIJhSWw5o
Video 2: https://youtu.be/NGsoqGoUONg

B. General questions - bold questions are verifiable truth

Robot

1. What is the name of Berlusconi ?
2. Could you describe me what do you usually eat at your home for christmas ?
3. Is it possible for a person to get burnt while using an oven ?
4. Can you tell me briefly what did you do last week-end ?
5. Can you describe what did you eat for breakfast this morning ?
6. In which city is located the Coliseum ?
7. What are the ingredients of your favorite dish ?
8. Could you describe me briefly the place where you live ?
9. What is the name of the chief town of Liguria region ?
10. Is Italy in Europe ?

Human

1. What is the name of your mother?
2. When did you graduated?
3.What do you during a typical day ?
4. Can an oven be hot?
5. What did you last new year eve ?
6. Which country would like to visit and why ?
7. Can you tell me what do you like to do during your free time ?
8. How do you cook pasta ?
9. Is the Coliseum located in Bari?
10. What language is spoken in the USA?

B. Video 1, 2 questions - bold questions are short type

Video 1 - Robot questions

1. Were there two or three people ?
2. Describe me the criminal act ?
3. Does the suspects look each other before to commit the criminal act ?
4. What is the age of the implicated ?
5. Describe me the behaviors of the suspects ?
6. Can you describe me the place where the robbery took place ?
7. Was there any violent acts committed ?
8. Describe me the aspect of the suspects ?
9. How the suspects were dressed ?
10. How many persons were present during the robbery ?

Video 1 - Human questions

1. Was there a woman?

2. How did they hide loot?

3. Was it crowded ?

4. Describe me how the suspects communicate between each other ?

5. Did they interact with someone ?

6. Describe me what they steal ?

7. Was someone else in the shop ?

8. Did they wear summer clothes ?

9. Which clothes did they wear ?

10. Did they have any particular aspect?

Video 2 - Robot questions

1. Was the criminal male ?

2. Describe me the criminal act ?

3. Was the person of color ?

4. Describe me what the criminal was wearing ?

5. How many people were present apart from the criminal ?

6. Tell me where did it happen ?

7. Was the criminal violent ?

8. What was approximately the age of the criminal ?

9. Describe me the physical aspect of the suspect ?

10. How did the criminal hide the loot ?

Video 2 - Human questions

1. Was the criminal female ?

2. How many people were in the shop during the robbery ?

3. Describe me the behavior of the suspect ?

4. Describe me the merchandise sell in the shop ?

5. Did the criminal had associate ?

6. Describe me what was robbed ?

7. Did the criminal have a backpack ?

8. Tell me what the other persons were doing during the robbery ?

9. Was the criminal wearing sporty Clothes ?

10. Describe me particular signs of the criminal ?
